# Combination therapy for bipolar disorder : What to combine and which cautions to take ?

**DOI:** 10.1192/j.eurpsy.2023.259

**Published:** 2023-07-19

**Authors:** M. Gardabbou, M. Maalej, R. Feki, I. Gassara, N. Smaoui, L. Zouari, J. Ben Thabet, S. Omri, N. Charfi, M. Maalej

**Affiliations:** psychiatry C department, Hedi Chaker, Sfax, Tunisia

## Abstract

**Introduction:**

Bipolar disorder is one of the leading causes of disability among young adults. Given the heterogeneity of the disorder and the complexity of its etiopathogenesis, combination therapy is often considered as part of the treatment regimen.

**Objectives:**

To assess the place of non-pharmacological interventions as a co-adjuvant to pharmacological treatment, to discuss the role of polytherapy in the management of bipolar disorder and to underline the drug to drug interactions to keep in mind.

**Methods:**

We present a critical review of recent international recommendations for the management of bipolar disorder. Two main evidence-based guidelines were included: The 2020 Royal Australian and New Zealand College of Psychiatrists clinical practice guidelines for mood disorders and The 2018 Canadian Network for Mood and Anxiety Treatment.

**Results:**

According to guidelines, the outcomes in bipolar disorder are improved when medication is combined with evidence-based psychological treatment and lifestyle changes. As to polytherapy, it is often recommended to maximise the treatment efficacy. Studies have shown that combination treatments tend to work faster and more effectively than monotherapy especially in episodes with higher index severity. For the management of agitation, an adjuctive treatment by Haloperidol with midazolam or promethazine can be prescribed. In acute mania, combination therapy with quetiapine, aripiprazole, risperidone or asenapine and lithium or divalproex is recommended as first-line treatment options. Combinations of mood-stabilizing drugs may be more often necessary when rapid cycling is present. In a manic episode with mixed features the use of divalproex with an atypical antipyshcotic is recommended. In bipolar I depression, lurasidone and lamotrigine are often used as adjunctive therapies. When anxious distress is present, the combination of olanzapine and fluoxetine has shown to be effective. In a depression with atypical features, tranylcypromine (IMAO) can be added to lithium, divalproex or a second generation antipsychotic for a better result. Adjunctive treatment of olanzapine with fluoxetine may be necessary in a depression with mixed features. However, in bipolar II depression and for maintenance treatment no adjunctive therapies are recommended. Finally, it is important to consider the adverse effects resulting from polytherapy. Using lithium as an adjunctive medication may increase the risk of tremor and acute dystonic reactions and can be a contributing factor for neuroleptic malignant syndrome, whereas divalproex can be an inducer or an inhibitor of some atypical antipsychotics.

**Conclusions:**

Rational polytherapy allows better and faster control over symptoms of bipolar disorder and should be considered after a detailed discussion of risks and benefits.

**Disclosure of Interest:**

None Declared

